# Coronary computed tomography angiography before percutaneous coronary intervention improves cardiovascular outcomes—a story too good to be true?

**DOI:** 10.1093/ehjimp/qyag063

**Published:** 2026-04-28

**Authors:** Anne Rebecca Schöber, Karl-Philipp Rommel

**Affiliations:** Department of Cardiology, University Medical Center of the Johannes Gutenberg University and DZHK, German Center for Cardiovascular Research, Partner Site Rhein/Main, Mainz 55131, Germany; Department of Cardiology, University Medical Center of the Johannes Gutenberg University and DZHK, German Center for Cardiovascular Research, Partner Site Rhein/Main, Mainz 55131, Germany

**This editorial refers to ‘Examining the association between cardiovascular outcomes in individuals undergoing percutaneous coronary intervention with or without a preceding coronary CT angiogram’, by K. Sawalha *et al*., https://doi.org/10.1093/ehjimp/qyag062.**

Percutaneous coronary intervention (PCI) is the cornerstone of coronary revascularization. Yet, despite advances in technology, adjunctive pharmacotherapy, and intravascular imaging, one of the remaining questions is: can better anatomical characterization before PCI improve outcomes after the procedure? Coronary computed tomography angiography (CCTA) has transformed the diagnostic work-up of patients with suspected coronary artery disease, improved risk stratification, and in some settings altered downstream management.^1–3^ Whether this translates into better outcomes among patients who eventually undergo PCI, however, is less clear. Sawalha *et al*.^4^ try to address this question by analysing a real-world cohort of patients undergoing *de novo* PCI with or without a preceding CCTA within 1 year.

The authors queried the TriNetX U.S. Research Network and identified adults undergoing PCI between 2013 and 2025. They excluded patients with acute coronary syndrome, or history thereof, prior PCI, or prior coronary artery bypass grafting. After 1:1 propensity score matching, 4936 patients remained in each group. The primary composite endpoint of all-cause mortality, myocardial infarction, or heart failure occurred less frequently in patients with pre-procedural CCTA, both at 1-year [9.6 vs. 13.0%; hazard ratio (HR) 0.74, 95% confidence interval (CI) 0.66–0.83] and at 5-years (16.1 vs. 23.7%; HR 0.79, 95% CI 0.72–0.86). The observed difference was mainly driven by lower rates of myocardial infarction and death, while heart failure was not significantly different at 1-year and only modestly lower at 5-years.

At first glance, these findings are clinically appealing and the concept is intuitive; an anatomical roadmap before PCI might improve lesion assessment, procedural planning, patient selection, and perhaps even the timing of invasive management. CCTA may also identify plaque burden and characteristics beyond the target lesion and trigger better preventive therapy. Such mechanisms are plausible, and they explain why this topic is of interest. With coronary imaging becoming increasingly sophisticated, the possibility that non-invasive imaging obtained before PCI could influence long-term outcomes is more than a technical possibility, it might enable the core of precision revascularization. The present analysis therefore addresses a relevant and timely clinical question.

However, this is also where enthusiasm must meet methodological sobriety. The major limitation of the study is not the propensity matching itself, but the variables it could not account for. Several clinically relevant factors were unavailable, including symptom burden, ischaemic severity, left ventricular function, frailty, rhythm, concomitant valvular heart disease, indication for PCI, lesion complexity, completeness of revascularization, and medication changes before and after imaging. This is the fundamental weakness of propensity score analyses: they can balance only what is measured. Even the most sophisticated adjustment cannot substitute for randomization when important baseline characteristics and risk-modifying variables are missing or incompletely captured. Yet these factors are central determinants of both the likelihood of undergoing CCTA and the risk of subsequent cardiovascular events. A patient referred for CCTA before PCI is therefore not simply a patient without CCTA plus one imaging test. Rather, such a patient may represent a different diagnostic pathway, a different degree of stability, a different threshold for invasive treatment, and potentially a different overall quality of care.

Accordingly, the study cannot be interpreted as showing that CCTA before PCI improves outcomes. Rather, it shows that patients who underwent CCTA before PCI had better outcomes. That distinction is not semantic, it is the central message. The mechanistic explanations offered, while reasonable and attractive, remains speculative in nature in the absence of randomized data.

The long inclusion period from 2013 to 2025 adds further complexity. PCI practice, secondary prevention, CT technology, and thresholds for both non-invasive and invasive testing have all evolved substantially over that time. Secular trends may therefore have favoured one group over the other in ways that matching cannot adequately capture.

And yet, despite all these limitations, the study should not be dismissed. It highlights a clinically important possibility: that the pathway leading to PCI may matter more than one might acknowledge. The value of this report may not lie in changing practice tomorrow, but in sharpening the questions we need to answer next. Which patients truly benefit from detailed anatomical non-invasive assessment before revascularization? Does pre-PCI CCTA improve lesion planning, reduce incomplete revascularization, alter adjunctive medical therapy, or merely identify a more stable phenotype? Are there specific subgroups, stable multivessel disease, ambiguous lesion morphology, diffuse atherosclerosis, in whom this approach is particularly informative? These are highly relevant questions, and the present analysis gives them momentum.

In the end, this study offers an interesting signal, not a finite answer. For now, CCTA before PCI should not be seen as outcome-modifying therapy. But neither should this association be ignored. Rather, it should be seen as a call to action to move beyond broad comparisons of imaging strategies and towards prospective studies that integrate anatomy, symptoms, physiology, procedural execution, and pharmacological optimization. Only then will we know whether pre-PCI CCTA is truly changing outcomes, or simply revealing who was destined to fate better in the first place.
